# Utility of Bulk T-Cell Receptor Repertoire Sequencing Analysis in Understanding Immune Responses to COVID-19

**DOI:** 10.3390/diagnostics12051222

**Published:** 2022-05-13

**Authors:** Hannah Kockelbergh, Shelley Evans, Tong Deng, Ella Clyne, Anna Kyriakidou, Andreas Economou, Kim Ngan Luu Hoang, Stephen Woodmansey, Andrew Foers, Anna Fowler, Elizabeth J. Soilleux

**Affiliations:** 1 Department of Health Data Science, Institute of Population Health, University of Liverpool, Liverpool L69 3GF, UK; h.kockelbergh@liverpool.ac.uk; 2 Department of Pathology, University of Cambridge, Cambridge CB2 1QP, UK; sce30@cam.ac.uk (S.E.); td417@cantab.ac.uk (T.D.); ermc2@cam.ac.uk (E.C.); knl25@cam.ac.uk (K.N.L.H.); stephen.woodmansey@mbht.nhs.uk (S.W.); 3 School of Clinical Medicine, University of Cambridge, Cambridge CB2 1QP, UK; ak2082@cam.ac.uk (A.K.); ae427@cam.ac.uk (A.E.); 4 Department of Respiratory Medicine, University Hospitals of Morecambe Bay, Kendal LA9 7RG, UK; 5 Kennedy Institute of Rheumatology, University of Oxford, Oxford OX3 7YF, UK; andrew.foers@kennedy.ox.ac.uk

**Keywords:** COVID-19, SARS-CoV-2, immune response, coronavirus, T-cell receptor repertoire, antibody, immunological memory, machine learning, diversity, immunoreceptor

## Abstract

Measuring immunity to severe acute respiratory syndrome coronavirus 2 (SARS-CoV-2), the causative agent of coronavirus disease 19 (COVID-19), can rely on antibodies, reactive T cells and other factors, with T-cell-mediated responses appearing to have greater sensitivity and longevity. Because each T cell carries an essentially unique nucleic acid sequence for its T-cell receptor (TCR), we can interrogate sequence data derived from DNA or RNA to assess aspects of the immune response. This review deals with the utility of bulk, rather than single-cell, sequencing of TCR repertoires, considering the importance of study design, in terms of cohort selection, laboratory methods and analysis. The advances in understanding SARS-CoV-2 immunity that have resulted from bulk TCR repertoire sequencing are also be discussed. The complexity of sequencing data obtained by bulk repertoire sequencing makes analysis challenging, but simple descriptive analyses, clonal analysis, searches for specific sequences associated with immune responses to SARS-CoV-2, motif-based analyses, and machine learning approaches have all been applied. TCR repertoire sequencing has demonstrated early expansion followed by contraction of SARS-CoV-2-specific clonotypes, during active infection. Maintenance of TCR repertoire diversity, including the maintenance of diversity of anti-SARS-CoV-2 response, predicts a favourable outcome. TCR repertoire narrowing in severe COVID-19 is most likely a consequence of COVID-19-associated lymphopenia. It has been possible to follow clonotypic sequences longitudinally, which has been particularly valuable for clonotypes known to be associated with SARS-CoV-2 peptide/MHC tetramer binding or with SARS-CoV-2 peptide-induced cytokine responses. Closely related clonotypes to these previously identified sequences have been shown to respond with similar kinetics during infection. A possible superantigen-like effect of the SARS-CoV-2 spike protein has been identified, by means of observing V-segment skewing in patients with severe COVID-19, together with structural modelling. Such a superantigen-like activity, which is apparently absent from other coronaviruses, may be the basis of multisystem inflammatory syndrome and cytokine storms in COVID-19. Bulk TCR repertoire sequencing has proven to be a useful and cost-effective approach to understanding interactions between SARS-CoV-2 and the human host, with the potential to inform the design of therapeutics and vaccines, as well as to provide invaluable pathogenetic and epidemiological insights.

## 1. Introduction

Severe acute respiratory syndrome coronavirus 2 (SARS-CoV-2), the causative agent of coronavirus disease 19 (COVID-19), has resulted in devastating global morbidity and mortality [1]. Significant progress has been made into understanding the immunology of COVID-19 and developing various vaccines that offer high levels of initial protection. However, the longevity of both infection- and vaccination-induced COVID-19 immunity remains to be determined [2]. T cells play an important role in the pathogenesis and resolution of the disease and may provide long-lasting immunity [2]. This review explores the advances in our understanding of T-cell immunity to SARS-CoV-2 achieved through bulk T-cell receptor (TCR) repertoire analysis. To assist readers in interpreting the published studies, we first discuss the methodologies that can be applied, before presenting biological insights obtained from TCR repertoire analysis. Information obtained via such studies has implications for the design of vaccines and therapeutics, as well as for epidemiology and patient-risk stratification.

## 2. Overview of T-Cells in COVID-19

The relationship between T cells and clinical outcome in COVID-19 is complex and incompletely understood [2]. The peripheral blood T-cell count shows an inverse correlation with disease severity and a high CD8:CD4 ratio is observed in mild cases, with a decreased CD8:CD4 ratio correlating with severe disease. The poorer disease outcomes in patients over 65 years old are likely due to the decreased size (or diversity) of the naïve T-cell pool. This, coupled with COVID-19-induced lymphopenia, causes a delayed or uncoordinated adaptive immune response and suboptimal viral clearance [3]. Despite marked lymphopenia in severe disease, a marker of poor prognosis, the number of SARS-CoV-2 specific T cells in peripheral blood is higher than that of mild and convalescent individuals [4]. Flow cytometry and ELISA-based analysis of secreted cytokines (e.g., IFNγ) demonstrate that SARS-CoV-2 specific T cells in severe disease show a restricted cytokine profile, and high expression of inhibitory surface receptors and proliferation markers [4]. In severe cases of COVID-19, T-cell responses are either insufficient, inappropriate, or excessive [3,4,5].

### 2.1. Immunological Memory and Longevity 

Understanding immunological memory is important for assessing the likelihood and severity of disease upon re-infection, as well as for epidemiological analysis and policy making. It is also a critical factor in determining how often booster vaccines should be offered to certain population subgroups, and clinically this may well be a potentially important role for T-cell immunity based tests in future. Evidence from SARS-CoV-1 suggests that T-cell immunity lasts for decades [6]. To date, antibody-based tests, such as lateral flow tests and ELISAs, have been used to infer immunity to SARS-CoV-2, but may not present a full picture. Neutralising antibody titres against the SARS-CoV-2 spike protein or receptor-binding domain decreased moderately over an 8-month follow-up period in 188 convalescent individuals. In contrast, 92% of individuals had detectable SARS-CoV-2-directed memory CD4+ T cells, as assessed by T-cell co-culture with SARS-CoV-2 peptides, followed by flow cytometric assessment of surface activation markers. The correlation between circulating antibody titres and T-cell immunity was incompletely understood [7]. Flow cytometry, using class I MHC tetramers and predicted optimal epitopes from SARS-CoV-2, demonstrated CD8+ specificity for SARS-CoV-2 in a higher percentage of subjects than antibodies, in a cohort of convalescent individuals, following mild or asymptomatic disease [8]. Furthermore, agammaglobulinaemic patients, who lack the ability to generate mature B cells or antibodies, can clear the SARS-CoV-2 infection without the need for mechanical ventilation, indicating that T cells may be sufficient to clear the infection with minimal symptomatic disease [3]. For some SARS-CoV-2 variants, such as the beta variant, against which Moderna (mRNA-1273) or Pfizer/BioNTech (BNT162b2) vaccine-induced antibody is relatively ineffective, as it is thought that a predominantly T-cell-mediated immune response still appears to confer immunity, at least decreasing disease severity and mortality [9,10,11]. It remains paramount to examine new variants that emerge for possible “T-cell escape” mutations. 

### 2.2. T-Cell Cross-Reactivity

Assessing T-cell responses as evidence of previous SARS-CoV-2 infection is confounded by T-cell cross-reactivity with seasonal coronaviruses, with a study demonstrating cross-reactive T cells against the spike protein of SARS-CoV-2 in 28% of healthy unexposed (pre-pandemic) donor samples [8]. One benefit of this relatively high level of cross-reactivity in the unexposed population is the potential for some pre-existing immunity to SARS-CoV-2 infection [12].

### 2.3. Pathogenic Effects of T Cells

T cells are a sensitive and long-lasting marker of immunity in mild cases, but are potentially pathogenic in severe cases. Multisystem inflammatory syndrome (MIS) has pathogenesis similar to toxic shock due to the Staphylococcal antigens TSS toxin 1 and enterotoxin B. It can affect all major body systems in both children and adults, manifesting as persistent fever and hyperinflammation. Cytokine storms, occurring in some adults with COVID-19 are thought to have a similar aetiology [5].

### 2.4. T-Cell Tests in Current Clinical Use

Current clinical tests for T-cell immunity include enzyme-linked immunosorbent spot (ELIspot) and intracellular cytokine staining, both methods presenting T-cell storage, transport, and handling challenges. Newer technologies include Adaptive Biotechnologies sequencing-based analysis of the TCR repertoire, which shows higher sensitivity than commercially available serological tests without apparently being confounded by responses to other pathogens [13].

## 3. The TCR Repertoire

Each T cell has a TCR; the sequence, and to some extent, the antigen specificity of which is essentially unique to that cell and its clones. The combined set of TCR sequences from all T cells in an organism is termed the TCR repertoire. Its characteristics, including diversity, composition and dynamics, are important indicators of immune responses to specific antigens and are powerful tools for the diagnosis and prognosis of immune-related diseases. 

**Figure 1:**
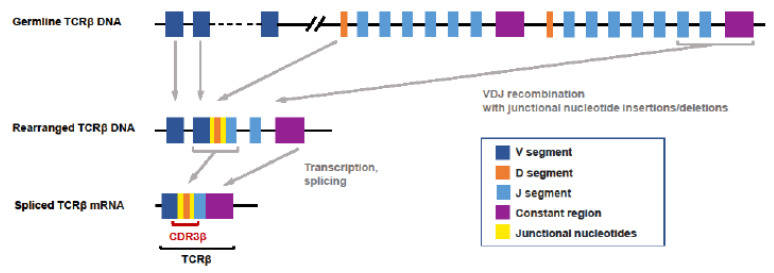
**V(D)J recombination determines T-cell receptor specificity.** The TCR specificity of αβ T cells is determined by the unique V(D)J recombination events that occur during the development of each T cell. During this process, V, D and J gene segments are randomly selected and are spliced together on the β chain, while the α-chain rearrangement of the V–J gene segments occurs in a similar process. During this process, the random addition or deletion of nucleotides can occur at segment junctions. The complementarity-determining region 3 (CDR3) encoded by sequences located in the V(D)J junction has the greatest diversity and is what determines the antigen specificity of each TCR. TCRβ: T-cell receptor beta; CDR3β: the gene sequence encoding the complementarity-determining region 3 of the TCR beta chain.

Similar sequences are present in cells with similar binding specificity, meaning that TCR repertoire sequencing can provide very useful insights into T-cell responses to a virus [14]. The mechanism by which unique TCR sequences are generated in each T cell is summarised in Figure 1. In brief, T cells, with receptors able to bind a very diverse array of antigens, contribute to the immunological protection of an individual. The genome would be unmanageably large if all unique DNA sequences required to encode these receptors were included in germline DNA [15,16]. TCRs are thus encoded in a combinatorial manner instead, with multiple separate DNA segments being brought together (Figure 1). This process occurs on two separate chains that are then paired, either as alpha-beta or gamma-delta, to present each T cell random, but unique, antigen specificity, which is not explicitly encoded by the genome. Subsequent positive and negative selections of T cells occur on the basis of the binding specificities of their receptors, removing in the thymus both those unlikely to bind antigen with appreciable affinity and those with autoimmune specificity, to leave a naïve mature T-cell repertoire. Because of the formation of junctions between germline-encoded gene segments at the variable positions, with potential template-independent insertion of bases, it can be appreciated that the genome will carry significant numbers of non-functional TCR rearrangements, for example, containing stop codons, due to shifts in the reading frame. Therefore, if sequencing is based on a DNA template, rather than RNA, it is important that non-functional TCR rearrangements are removed [17]. 

Upon meeting a cognate antigen, a naïve T cell would be activated, leading to clonal expansion and differentiation into effector cells [15,16]. Expansion of multiple closely related clones might thus be expected in response to a given antigen.

### 3.1. Sample Cohort Building for TCR Repertoire Analysis

When comparing immunoreceptor repertoires between disease groups, it is critical that cohorts are as large as possible and ideally age- and gender- matched, as age, in particular, is known to affect the TCR repertoire composition [18]. Potential genetic and environmental confounders should also be considered.

### 3.2. Laboratory Methods in ‘Bulk’ TCR Repertoire Sequencing

Next-generation sequencing (NGS) has unleashed the ability to analyse the sequence of large numbers of TCRs in parallel. All of the TCR sequences in one sample are known as the TCR repertoire. Two types of TCR repertoire analysis dominate current research: “bulk” population sequencing and single-cell sequencing. The focus of this review is on “bulk” sequencing, which provides information on the frequency of single-chain usage, presenting a high-resolution view of diversity and of clonal relatedness, as whole populations of cells can be sequenced at a time. However, in order to assess chain pairings (either alpha-beta or gamma-delta), single-cell sequencing is required, which is typically more expensive and captures a smaller number of cells. As such, many of the analytical challenges and biological insights available to single-cell sequencing of the repertoire are different to those available to bulk sequencing, and are not covered in this review.

#### 3.2.1. Substrate for Repertoire Sequencing

“Bulk” sequencing can be performed on DNA or RNA extracted from samples containing lymphocytes, most commonly blood, peripheral blood mononuclear cells (PBMCs) or pre-sorted lymphocytes and, less commonly, fresh frozen or formalin-fixed paraffin-embedded (FFPE) tissue. The advantages of blood or PBMC are non-invasive sample collection and availability of healthy control samples. DNA template has advantages over RNA, including greater stability and a 1:1 relationship between numbers of sequences and numbers of cells, rather than being confounded by transcript expression levels. For RNA, the number(s) of cells that had any given TCR sequence cannot be inferred. However, in our experience, a reasonably accurate assumption is that each unique complementarity-determining region 3 (CDR3) nucleic acid sequence comes from one cell only. In contrast, RNA has the advantage of only sequencing transcripts that are expressed, and thus likely to be functional (as explained in Figure 1), avoiding the need to screen out likely non-functional TCR sequences bioinformatically. 

#### 3.2.2. Methodological Considerations 

Commonly used methods for producing TCR repertoire libraries are summarised in Table 1. Methods are generally amplification-based, using either 5′RACE (Rapid Amplification of cDNA Ends) or multiplex polymerase chain reaction (PCR). More rarely, methods are based on hybridisation capture [19,20,21,22,23,24,25,26,27,28,29,30,31,32,33,34,35,36,37,38,39,40,41] (Table 1). Most methods use the Illumina sequencing platform, although some utilise the Ion Torrent [42,43,44] or Roche 454 methodologies [45,46]. Caution must be taken when comparing repertoire data generated using different immunoreceptor library preparation methods, because of method-specific bias towards certain V and J segments, which is mainly a consequence of the use of method-specific PCR primers [47]. While each method has individual strengths [48], a gold-standard approach would improve the integration of results across different studies. Irrespective of library preparation method, incorporating unique molecular identifiers (UMIs) into nascent reads assists in distinguishing PCR duplicates from clonal sequences, as well as avoiding artefacts arising from PCR bias or PCR/sequencing errors [32,47], although UMI incorporation can be challenging when working with multiplex PCR methods.

### 3.3. Analysis of “Bulk” TCR Repertoire Sequencing

The CDR3 sequences are of primary interest in classification analyses because CDR3 regions are the most diverse and directly interact with antigens [49]. Pre-processing of sequencing data before alignment may be required if UMIs are incorporated. Accordingly, sequencing reads in fastq or fasta format are aligned to a reference database of V(D)J segments (e.g., IMGT (www.imgt.org), GenBank (https://www.ncbi.nlm.nih.gov/nucleotide/)) and clonotypes are assigned on the basis of V-, D- and J-segment usage and CDR3 lengths and sequences [50], using tools, such as MiXCR [51], IgBLAST [52] and IMGT/HighV-QUEST [53], with these different bioinformatic methods producing substantially different outputs [54]. While bespoke computational approaches are used by some, various immune repertoire analysis platforms are available. For example, VDJtools [55] or VisTCR [56] can be used to understand the immune repertoire by calculation and visualisation of summary statistics, pertaining to some of the parameters described in Table 2. A similar platform, ARResT [57], combines IMGT/HighV-QUEST with the analysis and visualisation of the resulting immune repertoire data. The adaptive immune receptor repertoire community (AIRR-C) is one of a number of repositories for COVID-19 TCR repertoire data [58]. 

#### 3.3.1. Principles of Analysis of CDR3 Sequences

Multiple different analyses are possible (Table 2 and Table 3), including simple descriptive analyses (CDR3 length, V(D)J-segment usage, amino acid proportions), identification of clonotypes and their neighbours, more complex mathematical analyses of diversity, richness and evenness (Table 2), identification of specific motifs (Table 3), and machine learning methods. However, all these methods are subject to amplification bias, the type of starting material (DNA or RNA) and the quality and number of cells in the starting material.

#### 3.3.2. Clonotypic Analysis

Simple analysis, such as CDR3 length profiles and V(D)J usages (Table 2), can be very effective at identifying clonality in samples. However, individual sequences are not considered and fine details may be obscured within these statistics. Clonotypic frequencies [50] provide a more in-depth perspective, and may vary between different patient groups, or between patients with a particular condition and controls. Any highly abundant receptors are assumed to be part of an active immune response whilst receptors that are observed in samples from multiple individuals with similar clinical conditions are thought to be capable of binding to a shared antigen [17]. Clonotypic analysis may focus on identifying known COVID-19-associated TCR sequences [49]. Analysis restricted only to clonotypically identical sequences may be too strict, particularly when considering the repertoires of different patients [17], meaning that motif-based analysis may provide more biologically meaningful information. 

#### 3.3.3. Diversity Profiling and Related Analyses

TCR diversity is a measure of the numbers of different CDR3 clonotypes in a sample and can be measured in multiple ways (Table 2) [17,49,59,60,61,62,63,64,65,66,67,68,69,70,71,72,73,74,75]. Diversity measures adapted from ecology have commonly been used to characterise the TCR repertoire, particularly Shannon diversity, Simpson diversity and Hill diversity [67,74,76]. In the context of repertoire profiling, they describe TCR clonotype abundance, richness (number of unique clones) and evenness (the degree to which different clonotypes are equally represented in the sample). The different measures place different levels of importance on clonal characteristics, for instance, Simpson diversity is more sensitive to clonal dominance, whereas Shannon diversity is more sensitive to rare clonotypes. Although Pielou’s evenness index provides a more thorough description of repertoire structure than Simpson or Shannon diversities, none of these measures looks at individual sequences, but rather aim to characterise the repertoire as a whole.

**Table 2:** Diversity-based methods of TCR repertoire analysis.

Analytical Approach	Principles/Interpretation
CDR3 length profiles [59]	Assumed to be Gaussian distributed Clonal expansions/depletions skew the distribution
VDJ usage	Over/under representation of specific gene segments an indication of immune response
Clonal abundance	Number of times a clonotype appears in a sample (ideally excluding PCR duplicates) Identify highly abundant sequences as clonal
Clonal frequency	Percentage of CDR3 sequences represented by a specific clonotype (excluding PCR duplicates) Identify sequences that comprise a large percentage of all the repertoire as clonal
Richness [62]	Total number of unique clonotypes High or low numbers of unique clonotypes indicative of immune irregularities
D50 diversity [64]	Minimum percentage of unique clones amounting to 50% of the total sequences Low percentage indicative of low diversity and clonality
Simpson diversity [65,66,67]	Estimates the probability of any two randomly sampled TCRs having different clonotypes Clonal populations have high values
Shannon diversity [61,67,69,70,71]	Assesses the richness and unevenness of a TCR repertoire, the number of clonotypes and differences in their frequencies Higher values denote a more diverse clonotype distribution
Hill’s diversity (Hill’s evenness) [73]	Describes the effective number of clonotypes within a sample
Pielou’s evenness index [64]	Shannon diversity index divided by maximum possible Shannon diversity index Indicates the degree to which different clonotypes are equally represented in the sample
Parametric methods [74]	Assume underlying distribution of TCR clones, commonly Poisson or Zipfian Broad properties of the repertoire inferred from fitted model parameters

#### 3.3.4. Analyses Based on Sequence or Motif Identification

While analysing TCR metrics, such as the abundance of T cells, unique CDR3 sequences, or entropy, can provide an assessment of the diversity of the repertoire or its level of clonal expansion, these metrics are sequence agnostic. They are unable to assess the antigen-specific nature of the repertoire, and, furthermore, cannot identify antigenic associations with clinical outcomes in datasets. Clustering methods aim to group together TCR sequences that are either clonally related, having very similar CDR3s, or which likely bind the same antigen, having conserved motifs or similar physiochemical properties (Table 3). In identifying the clusters related to a particular disease, some methods use the cluster frequency or over-representation relative to a control group. It should be noted that the identification of such motifs is computationally demanding due to the vast number of combinatorial possibilities, and the methods presented in Table 3 often have a speed trade-off as the complexity of the algorithm and the patterns identified increase. Any identified motifs still require some form of validation, either in an independent test set or experimentally, before their real clinical utility can be exploited. Notwithstanding, multiple approaches have been taken to predict the likely antigens bound, including NetTCR, TCRex and MIRA, and databases, such as VDJdb and McPAS-TCR have been created to facilitate the sharing of known antigen specificities of TCR sequences [77,78,79,80,81].

**Table 3:** **TCR clustering methods.**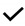 indicates that the TCR clustering method uses the feature to define a cluster.

Method	Features							
	V(D)J Alignment	CDR3s	Short Motifs	Physio-Chemical Properties	Amino Acids	Nucleotides	Frequency	Enrichment
GIANA [82]	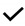 (V Only)	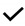		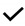	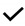			
ALICE [83]		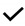			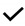		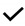	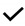
clusTCR [84]		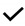		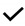				
GLIPH2 [85]		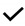	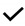	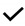	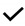			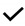
iSMART [86]		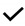	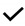	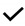	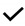			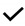
TCRdist [87]	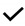 (CDR1 and 2)	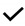			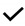			
TCRNET [83]	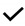 (V and J)	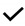	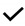					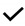 (control samples required)
ImmunoMap [88]		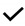		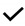			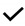	
MiXCR [51]	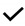 (V and J)	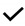				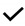		

#### 3.3.5. Machine Learning to Predict Diagnosis, Exposure to Infection or Outcome of Infection

**Figure 2:**
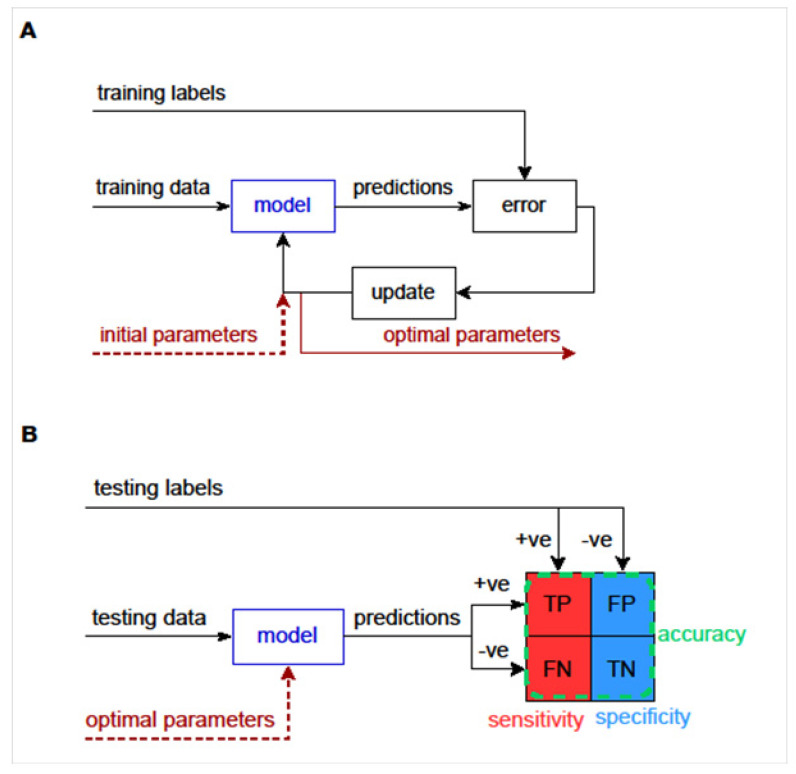
**Overview of machine learning approaches.** (**A**) Training data and training labels are used to train the model by obtaining its optimal parameters. The model with initial parameters makes predictions for the training data. These predictions are compared to the training labels and the error between them is calculated. The model parameters are updated to correct for the error. These steps continue until the error cannot be made smaller and the model is trained. (**B**) A trained model should be tested with a separate set of data and labels called the testing data. Positive or negative predictions are made for the testing data using the trained model with its optimal parameters. Each prediction is compared to the positive or negative testing label and categorised as a true positive (TP), false positive (FP), true negative (TN) or false negative (FN). The model’s sensitivity can be estimated as TP/(TP + FN), specificity as TN/(TN + FP) and accuracy as (TP + TN)/(TP + FP + TN + FN).

Machine learning algorithms (artificial intelligence, AI), which can be trained using examples, may be applied to produce TCR repertoire-based diagnostic models that can classify samples by diagnosis (e.g., COVID-19 unexposed versus previously COVID-19 infected). Such models can be built without the knowledge of the specificities of TCRs driving the classification and are typically supervised, in that they are provided with data from samples with a known label. Training can involve the selection of model parameters that produce the most accurate classification (Figure 2A). Such classification may be performed on the basis of closely related full-length CDR3 sequences [89], permitting 1 to 2 amino acid substitutions, often with weightings for the relatedness of substituted amino acids [90]. Alternatively, grouping may be performed on the basis of shorter motifs that form part of CDR3 sequences [14]. Testing then involves applying these optimal model parameters to a new set of testing data, in order to determine the classification accuracy (Figure 2B).

To present an example, we successfully used clustering combined with a supervised training approach [17], to construct a classifier to separate samples donated by COVID-19 convalescent individuals from COVID-19-naïve individuals [14]. In our approach, CDR3 sequences were broken into contiguous amino acid sequences of length k (kmers), so that CDR3s that were non-identical, but shared a short motif, could be considered similar. The kmer length that provided the optimal classification was identified during the training stage [14]. Although this model for previous COVID-19 infection performed well in leave-one-out cross-validation, it has not been tested on any independent test sets analysed with the same laboratory methodology, due to small cohort sizes. The generalisability of this classifier between datasets thus remains unknown. A CDR3-based machine learning method, i-CAT, was also recently described to be able to separate TCR repertoires from individuals post-SARS-CoV-2 infection from unexposed individuals. However, the sample cohorts were exceptionally small, meaning that overfitting may have occurred, producing a falsely high accuracy [89]. The machine learning algorithm, DeepTCR, is a multiple-instance deep learning repertoire classifier assessing a combination of CDR3 sequence and V/D/J gene-segment usage [91], which has been used successfully to predict patients with severe versus milder SARS-CoV-2 infection from their repertoire sequencing [92]. However, it did not generalise between two separate cohorts, likely due to geographical and demographic differences, although overfitting could not be excluded. DeepTCR includes a convolutional neural network and is a platform for deep learning that can be applied at the level of individual TCR sequences or the whole TCR repertoire. It can learn patterns in the data that may be used for both descriptive and classification/predictive purposes [91]. Most Machine learning approaches are currently limited by the sample size. Although combining data from multiple sources could improve the robustness of these models with an enlarged training dataset, it is important to keep in mind the limitations of doing this due to different TCR sequencing methods, library preparation and target enrichment. However, datasets are increasing in size and, therefore, we expect machine learning-based classification models to increase in their utilities.

#### 3.3.6. Machine Learning to Identify New Antigen-Specific Sequences 

Machine learning has previously been used for the identification of novel sequences in TCR repertoires [93] and this has also been attempted for SARS-CoV-2. For example, using DeepTCR, 25 sequences most predictive of severe COVID-19 infection were identified. Multiplex Identification of T-cell Receptor Antigen Specificity (MIRA) was applied to these sequences and SARS-CoV-2 antigen specificity was predicted and shown to differ (a) between CD4 and CD8 T cells and (b) between individuals with mild and severe disease. As a consequence of this approach, it was possible to construct an epitope-specific classifier to predict whether patients had mild or severe disease [92].

## 4. Biological Insights into COVID-19 from T-Cell Receptor Analysis

**Figure 3:**
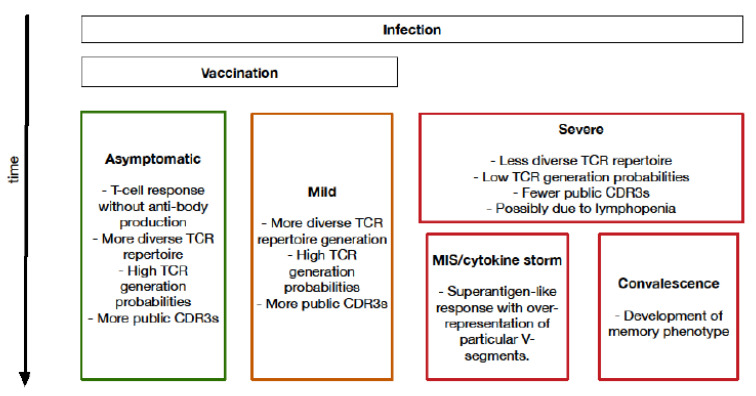
**Schematic overview of insights into TCR repertoire, observed over time after infection, obtained by bulk TCR repertoire sequencing.** MIS: Multisystem Inflammatory Syndrome.

A broad range of biological insights have been gained from bulk TCR repertoire sequencing in COVID-19. Apart from the specific TCR sequences identified, which are contained in the various publications we cite, an overview of many of the biological findings can be observed in Figure 3 and summarised in Table 4. These publications were identified through a PuBMed search for papers containing the terms ‘T-cell receptor [TCR] sequencing’ or ‘T-cell receptor [TCR] repertoire’, and ‘COVID-19’ or ‘SARS-CoV-2’ in their title or abstract. Papers were then manually screened for their use of bulk TCR sequencing. Whilst these papers all represent interesting results, many of these studies are limited in their sample sizes, availability of healthy controls or pre-pandemic samples and lack of HLA typing.

### 4.1. Relative Contributions of T Cells and B Cells to SARS-CoV-2 Immunity

Shomuradova and colleagues showed that healthy donors during the pandemic had increased numbers of SARS-CoV-2-specific T cells, but not antibody response, likely indicating either prior asymptomatic SARS-CoV-2 infection or the presence of pre-existing cross-reactive T cells that represented a response to previous infection with a related virus [94]. Furthermore, in the same study, some convalescent patients had anti-SARS-CoV-2 TCRs, but no detectable antibody response after a certain period post-infection. Our own analysis of the convalescent cohort that had had mild infection in Schultheiss and colleagues’ study [95] showed an ability to predict SARS-CoV-2-immunity from TCR, but not BCR, repertoire data [14]. This indicates that there are fewer common features of BCR than TCR repertoire data between previously SARS-CoV-2 infected individuals, which may be because a SARS-CoV-2-specific B-cell response was limited in strength and duration or absent in a proportion of the individuals studied. 

### 4.2. Association between Higher Repertoire Diversity and Improved Outcomes

Multiple studies have shown that, in mild infections with SARS-CoV-2, the TCR repertoire remains relatively diverse, with high generation probability (i.e., broadly predictable from germline rearrangement patterns) TCR sequences persisting. This indicates that the repertoire does not simply consist of T cells responding to a specific antigen. Furthermore, the frequencies of specific clonotypes, even those that are SARS-CoV-2-specific, are not particularly high, in contrast to findings in severe COVID-19, in which there are smaller numbers of more frequent SARS-CoV-2-specific sequences. Notwithstanding, a broad range of SARS-CoV-2-specific sequences are seen in mild disease, with many CDR3 sequences shared between multiple individuals, i.e., public CDR3 sequences [92,94,95,96,97]. One caveat is the fact that younger age may confound this observation. This is because younger age is associated both with milder COVID-19 disease and with broader TCR repertoires than in older people [18]. In both mild and severe disease, the TCR repertoire in peripheral blood increases in diversity during convalescence. Asymptomatic infection is thought to follow a similar course, in terms of TCR repertoire profiles, to mild disease [94]. 

### 4.3. Kinetics of CD4 and CD8 T-Cell Responses

CD4+ and CD8+ T-cell clonotypes both undergo transient clonal expansion after infection, with similar kinetics, with clonal contraction after day 15 and the majority acquiring effector memory phenotypes by day 30. A study of only 2 patients showed a separate episode of T-cell expansion on days 15–37 after the infection [98]. While this may have been due to priming of more T cells by antigen-specific B cells, migration of SARS-CoV-2-specific T cells from lymphoid organs or bystander activation of non-SARS-CoV-2 specific T cells, the possibility that this was due to triggering by another infection cannot be excluded, and so this second wave of CD4/CD8 T-cell expansion requires corroboration in other larger studies. 

### 4.4. Importance of Specific V-, D- and J-Segment Usage

Few studies have found a very strong association between particular V-, D- and J-segment usage and prior exposure to SARS-CoV-2. However, in patients with a severe/hyperinflammatory COVID-19 clinical picture, four TCR Vβ gene segments (TRBV5-6, TRBV14, TRBV13 and TRBV24-1) were found to be overrepresented with little Jβ gene-segment skewing [5], suggesting a selective pressure preferentially acting on V-segment distribution. The same paper also used computational models and demonstrated that the spike protein of SARS-CoV-2, in contrast to other coronaviruses, exhibits a high-affinity motif for binding TCRs and may form a ternary complex with MHC-II, permitting it to behave similar to a superantigen, such as staphylococcal enterotoxin B. This provides a possible explanation for SARS-CoV-2 causing a cytokine storm in some adults and multisystem inflammatory syndrome in children and some adults.

### 4.5. Importance of SARS-CoV-2 Specific TCR Sequences and Motifs

TCR specificity was predicted in some of the datasets shown in Table 4 in one of two ways. One method is to undertake a functional assay, such as T-cell stimulation assays with subsequent flow cytometric analysis of cell surface phenotype and assaying T cells for their ability to bind to a fluorescently labelled MHC tetramer refolded with a selected SARS-CoV-2 peptide antigen [94,95]. The TCR repertoires of these likely SARS-CoV-2-specific T cells can thus be sequenced and analysed. The second approach is prediction by analogy to TCR sequences, the specificity of which is already known. The Multiplex Identification of the T-cell Receptor Antigen Specificity (MIRA) platform [80] was used for this purpose in several studies [92,98,99]. Between these two approaches, numerous potentially SARS-CoV-2-specific TCR sequences were identified.

**Table 4:** Biological insights into COVID-19 from bulk TCR repertoire sequencing.

First Author	Number of Samples	Cells	DNA/RNA	Loci	Key Points
Chang [97]	- 3 COVID-19 patients with mild disease - 6 with pneumonia	PBMCs	RNA	TRB	SARS-CoV-2-associated TCR clusters exhibited significantly higher TCR generation probabilities and most were public compared to those from pneumonia Different patterns of CDR3 sequence motifs in SARS-CoV-2-associated TCR clonotypic clusters.
Cheng [5]	- 38 patients with mild to moderate COVID-19 disease - 8 patients with severe disease (drawn from the Schultheiss cohort [95] described below)	PBMCs	DNA	TRB	Four TRB gene segments were overrepresented in severe COVID-19 patients. Computational models demonstrated that the spike protein of SARS-CoV-2, exhibits a high-affinity motif for binding TCRs and may form a ternary complex with MHC-II, permitting it to behave similar to a superantigen, such as staphylococcal enterotoxin B.
Minervina [99]	- 2 patients post mild COVID-19 - 5 time points	CD4+ and CD8+ T cells	RNA	TRB TRA	CD4+ and CD8+ T-cell clonotypes undergo transient clonal expansion after infection, with similar kinetics, the majority acquiring memory phenotypes, with clonal contraction after day 15. By day 30 post-infection, most pre-infection central memory clones were detected in the effector memory subpopulation.
Niu [96]	- 10 patients, early stage to recovery COVID-19 - 4 time points - 15 healthy controls	PBMCs	RNA	TRB TRA TRG TRD	Low number of TCR sequencing reads in early disease with gradual increase in clinical improvement, especially during convalescence, when some dominant clones remained. Number of TRB sequencing reads increased to the same level as healthy controls after recovery.
Rajeh [89]	- 6 patients post-COVID; 2 time points - 4 pre-pandemic controls	PBMCs	RNA	TRB TRA	100% classification accuracy achieved in predicting previous SARS-CoV-2 infection and thus likely immunity.
Schultheiss [95]	- 19 patients recovered from mild disease - 20 patients with active infection, severe disease - 39 age-matched healthy controls	PBMCs	DNA	TRB	150 clonotypic clusters in COVID-19 patients were identified that are likely of pathophysiological relevance. The longitudinal monitoring of one patient during active disease and recovery identified clonotypes that expanded during the patient’s successful immune response towards SARS-CoV-2. These clonotypes encompassed amino acid motifs that were also shared by other patients at recovery. T-cell repertoires of patients with a mild clinical course who recovered from COVID-19 were highly diverse.
Shomuradova [94]	- 34 recovering patients - 2 time points - 14 healthy donors - 20 pre-pandemic controls	CD4+ and CD8+ T cells	RNA	TRB TRA	Healthy donors during the pandemic had increased numbers of SARS-CoV-2-specific T cells, but no antibody response, likely indicating prior asymptomatic infection or activation of pre-existing immunity. Some convalescent patients had anti-SARS-CoV-2 TCRs, but no detectable antibody response. In convalescent patients, there was a public and diverse, high Pgen T-cell response to SARS-CoV-2 epitopes. CD4+ and CD8+ T-cell responses to the spike protein were mediated by groups of homologous TCRs, some of them shared across multiple donors. Hundreds of TCR motifs/ clonotypic clusters were identified, 25 of which were shared across multiple donors. For 19 of these, a potential cognate epitope or restricting HLA allele could be predicted.
Shoukat [14]	- 19 patients recovered from mild disease - 39 age-matched healthy controls - Obtained from Schultheiss [95].	PBMCs	DNA	TRB	Accurate sample classification possible on the basis of TCR repertoires (training accuracy 96.4%; validation accuracy 92.9%), but not BCR repertoires (training accuracy 74.5%; testing accuracy 47.3%).
Sidhom [92]	- 179 patients with mild disease - 106 patients with severe disease - Drawn from the ImmunoCode database	PBMCs	DNA	TRB	Total T cells, total nucleic acid template and total numbers of rearrangements were lower in severe versus mild infection, during the peak of infection. The 25 most predictive sequences for severe infection contained amino acids most predictive of disease severity in the central part of the CDR3 sequences. Using MIRA, specific SARS-CoV-2 antigen specificity was predicted and shown to differ (a) between CD4 and CD8 T cells and (b) between individuals with mild and severe disease. Able to construct an epitope-specific classifier to predict whether patients had mild or severe disease.
Swanson [99]	- 233 vaccinated patients; samples collected pre- and post-vaccination	PBMCs	DNA	TRB	A significant increase in the fraction of total T cells and fraction of unique TCRs that were spike protein-specific 28 days post second dose. Breadth and depth increases were comparable to COVID-19 convalescent patients. CD4 responses mapped to a broad range of parts of the spike protein, but CD8 responses were more restricted, most likely due to HLA restriction.
Wang [100]	- 9 patients of 2 weeks convalescence - 20 patients of 6 months convalescence - 5 healthy controls	CD4+ and CD8+ T cells	RNA	TRB TRA	TRBV6-5-TRBD2-TRBJ2-7 is the most enriched V(D)J gene-segment combination in both CD4+ and CD8+ T cells among patients. Identified identical CDR3 motifs of the TRA and TRB from CD8+ T cells that are significantly enriched in convalescent patients.
Hu [101]	- 5 recovered volunteers at least 14 days post recovery - 5 unexposed healthy donors	PBMCs	RNA	TRB	Identified 6 prevalent CDR3 amino acid patterns among sorted CD8+ T cells in recovered patients. No cross-reactive memory T cells identified in unexposed healthy donors.
Simnica [102]	- Blood samples: - 140 from unrelated COVID-19 patients - 140 pre-pandemic age-matched controls - Brain tissues: - 5 deceased patients with COVID-19, 40 brain tissue sections	PBMCs Brain-derived T cells;	DNA	TRB	Previously reported public TCRs in COVID-19 patients shown to have only slightly higher frequencies in COVID-19 patients than that of unexposed controls. 68 different clonotypes identified from brain-derived T cells of COVID-19 patients, which have a public nature and have potential for diagnosis.
Shimizu [103]	- 19 unexposed healthy donors with cross-reactive CD8+ T cells	CD8+ T cells	DNA	TRB TRA	Identified the immunodominant S-protein epitopes of SARS-CoV-2 that is responsible for the activation of cross-reactive CD8+ T cells in HLA-A24 people who have not been exposed to SARS-CoV-2.
Li [104]	- 54 COVID-19 patients in different phases (asymptomatic, symptomatic, convalescent, and re-detectable positive cases) - 16 healthy donors	PBMCs	RNA	TRB	Identified unique V–J-gene usage in asymptomatic and re-detectable positive cases. No HLA haplotype was found to be significantly correlated with disease stages.

### 4.6. Vaccine-Induced T-Cell Responses in Comparison to Responses to Native Infection

TCR repertoire responses to the AstraZeneca AZD1222 COVID-19 vaccine show similar changes to mild infection [99]. A total of 233 participants were vaccinated with AZD1222 or the MenACWY vaccine as a control, with doses approximately 4 or 12 weeks apart. Post-vaccination, participants had a significant increase in the fraction of total peripheral blood T cells and fraction of unique TCRs that were spike protein-specific 28 days after the second vaccine dose, with a similar depth and the breadth of the responses regardless of the dosing schedule. The breadth and depth increases were comparable to COVID-19 convalescent patients and no increase in TCR breadth of non-spike protein-specific SARS-CoV-2 TCRs was observed. As seen in SARS-CoV-2 infection [92], post-vaccination CD4 T-cell responses could be mapped to a broad range of parts of the spike protein, but CD8 responses were more restricted, most likely due to HLA restriction [99]. Peer-reviewed publications containing TCR repertoire data for other vaccines are awaited.

## 5. Areas for Further Research

Significant inroads have been made into understanding COVID-19 by means of analysis of TCR repertoire data, but much remains to be done. Further integration with single-cell data, in which chain pairing is known, will provide new insights, as will the ability of additional binding or functional studies to delineate new SARS-CoV-2-specific TCR sequences. Detailed mapping of particular TCR sequences to SARS-CoV-2 provides a tool kit for understanding the likely impact of new SARS-CoV-2 mutations upon largely vaccinated populations. While decreased TCR diversity acts as a useful biomarker to predict poorer prognosis, further identification of specific TCR sequences that are associated with unfavourable outcomes is desirable. In the context of vaccination, it is important to understand the risk of COVID-19 infection and/or severe disease post-vaccination as a function of the presence of specific TCR sequences. This requires large longitudinal studies of TCR repertoires in vaccinated individuals. 

## 6. Conclusions

T cells form the backbone of the immune system and it is, therefore, of little surprise that they play such critical roles in determining the outcomes of infection with COVID-19. T cells carry natural “barcode” sequences, by virtue of their TCR variable-region sequences, particularly the CDR3 component. This gift of nature has provided the opportunity to study T cells in great detail and to begin answering key questions about the course and longevity of infection or vaccine-induced immunity to SARS-CoV-2.

## Figures and Tables

**Table 1:** Principles of laboratory methods for TCR repertoire sequencing.

Method	DNA/RNA	Principles	Advantages	Disadvantages	Examples of Manufacturers
5′RACE	RNA	- Extra 5′ dCTP nucleotides are added to the sequence during cDNA synthesis using a modified Moloney Murine Leukaemia Virus (MMLV) reverse transcriptase allowing 3′ dGTPs and 5′ adaptor incorporation [33,34,35]. - The cDNA undergoes two rounds of PCR using one 5′ adaptor sequence and on 3′ TCR constant region primer [33,34,35]. - NGS adaptors are added by ligation or are incorporated into primers during the second round of PCR [33,34,35].	Using only one set of oligonucleotides per PCR reaction greatly reduces PCR bias, while the 3′ TCR constant region primer significantly increases specificity [19]. The addition of molecular barcoding (unique molecular indices or UMIs), together with the adaptor, helps to correct amplification bias at the analysis stage [30,31,32].	Relatively complex workflow.	iRepertoire, Clontech/Takara, MiLaboratories
Multiplex PCR	DNA, RNA	- DNA:NGS adaptors can be incorporated into the original multiplex primers to make it a one-step process. - RNA: An initial cDNA synthesis step is performed using either non-specific primers [24,25] or a targeted primer to the RCR constant region [26,27] - The multiplex PCR is then performed using multiple primers for each known V and either J (genomic DNA (gDNA)) or C (cDNA) region [28]. - Some methods a second round of PCR is used to add NGS adaptors	Simple pipeline for DNA; slightly more complex for RNA. A one-step PCR process can generate a library if adaptors (+/− UMIs) are included in the primers [20,21,22,23]. Multiplex PCR amplification and Illumina-based NGS are also offered by some companies, such as Adaptive Biotechnologies and Invivoscribe Inc., as a service [20,21,22,23].	The J region rather than the C region is used for the reverse primer for gDNA. Due to the large intron found between these regions [29] and the large number of J gene segments, the use of multiple J primers may increase amplification bias due to preferential binding and amplification [25], which can distort the entire TCR repertoire. UMI incorporation is also more difficult than for 5′RACE.	Adaptive Biotechnologies (Immunoseq); Invivoscribe (Lymphotrack)
Hybridisation capture	DNA, RNA	- Genomic DNA or cDNA derived from mRNA require an initial fragmentation/ sonication step [36,37,38]. - End repair/A tailing is followed by ligation to adaptors with or without UMIs [37,38,39]. - A set of specialised biotinylated complementary oligonucleotide baits, specific for the locus of interest, is hybridised to the DNA library, permitting capture of the target sequence [37,38,39,40]. - A final round of PCR is done to release the library from the baits [37,38,39,40].	Easy addition of UMIs, makes it a very powerful process to analyse BCR/TCR repertoire and has even shown promise in allowing more than one locus to be targeted in one reaction [41]. Likely to avoid PCR bias.	Relatively complex workflow. Risk of capturing many unrearranged sequences.	Bespoke approaches
